# Dysbiosis of the intestinal microbiota in neurocritically ill patients and the risk for death

**DOI:** 10.1186/s13054-019-2488-4

**Published:** 2019-05-31

**Authors:** Ruoting Xu, Chuhong Tan, Jiajia Zhu, Xiuli Zeng, Xuxuan Gao, Qiheng Wu, Qiong Chen, Huidi Wang, Hongwei Zhou, Yan He, Suyue Pan, Jia Yin

**Affiliations:** 1 Department of Neurology, Nanfang Hospital, Southern Medical University, Guangzhou, China; 2 State Key Laboratory of Organ Failure Research, Microbiome Medicine Center, Division of Laboratory Medicine, Zhujiang Hospital, Southern Medical University, Guangzhou, 510282 China

**Keywords:** Gut microbiome, Dysbiosis, Critical illness, Stroke, Prognosis, Biomarker, Cohort study

## Abstract

**Background:**

Despite the essential functions of the intestinal microbiota in human physiology, little has been reported about the microbiome in neurocritically ill patients. This investigation aimed to evaluate the characteristics of the gut microbiome in neurocritically ill patients and its changes after admission. Furthermore, we investigated whether the characteristics of the gut microbiome at admission were a risk factor for death within 180 days.

**Methods:**

This prospective observational cohort study included neurocritically ill patients admitted to the neurological intensive care unit of a large university-affiliated academic hospital in Guangzhou. Faecal samples were collected within 72 h after admission (before antibiotic treatment) and serially each week. Healthy volunteers were recruited from a community in Guangzhou. The gut microbiome was monitored via 16S rRNA gene sequence analysis, and the associations with the clinical outcome were evaluated by a Cox proportional hazards model.

**Results:**

In total, 98 patients and 84 age- and sex-matched healthy subjects were included in the analysis. Compared with healthy subjects, the neurocritically ill patients exhibited significantly different compositions of intestinal microbiota. During hospitalization, the α-diversity and abundance of *Ruminococcaceae* and *Lachnospiraceae* decreased significantly over time in patients followed longitudinally. The abundance of *Enterobacteriaceae* was positively associated with the modified Rankin Scale at discharge. In the multivariate Cox regression analysis, *Christensenellaceae* and *Erysipelotrichaceae* were associated with an increased risk of death. The increases in intestinal *Enterobacteriales* and *Enterobacteriaceae* during the first week in the neurological intensive care unit were associated with increases of 92% in the risk of 180-day mortality after adjustments.

**Conclusions:**

This analysis of the gut microbiome in 98 neurocritically ill patients indicates that the gut microbiota composition in these patients differs significantly from that in a healthy population and that the magnitude of this dysbiosis increases during hospitalization in a neurological intensive care unit. The gut microbiota characteristics seem to have an impact on patients’ 180-day mortality. Gut microbiota analysis could hopefully predict outcome in the future.

**Electronic supplementary material:**

The online version of this article (10.1186/s13054-019-2488-4) contains supplementary material, which is available to authorized users.

## Background

The crosstalk between the host and intestinal flora strongly influences host physiology in health and disease. However, the understanding of the microbiome is still in its infancy. Recently, many reports have revealed that changes in the gut microbiota are related to myasthenia gravis [1], Parkinson’s disease [2] and critical illness [3]. Severe insults to the gut have been hypothesized to promote multiple organ dysfunction syndrome as a result of disruption of commensal bacteria [4]. In previous studies, a low microbial diversity has been associated with an increased risk of mortality in critically ill patients [5, 6], and domination by certain pathogens has been identified as an independent risk factor for adverse outcomes [7–9]. Neurocritical care in the management of acute neurologic injury has rapidly evolved over the last 30 years [10]. However, little information is available regarding the ways in which the microbiota changes during neurocritical illness and whether the features of the intestinal microbiome are associated with mortality in the setting of neurocritical care.

The implementation of prebiotics [11], probiotics [12] or faecal transplant [13] to restore the “health-promoting” microbiome suggests that gut microbiota modulation can be used to monitor or cure disease [14–16]. However, the composition and community structure of microbes that colonize the human gut during critical illness have not been fully understood. Advances in next-generation sequencing provide opportunities to determine the gut microbiome composition in individuals as “microbial fingerprints”. Therefore, additional knowledge of the characteristics of the gut microbiota in neurocritically ill patients is required to better understand the microbial disturbances associated with clinical outcomes to identify patients who would benefit from microbiota-targeted therapies [14].

In this study, we characterized the phylogenetic composition of the faecal microbiota in 98 patients suffering from neurocritical illness using 16S rRNA gene sequence analysis and performed a subgroup analysis in patients with critically ill stroke. We also examined whether the abundances of specific taxa in the intestinal microbiota were associated with mortality within 180 days of admission.

## Methods

### Subjects

This study was a prospective observational study conducted in the neurological intensive care unit (neuroICU) of Nanfang Hospital, a university-affiliated academic hospital in Guangzhou, China, from December 2016 to February 2018. Adult patients admitted to the neuroICU with a Glasgow Coma Scale (GCS) of < 11 and an expected length of intensive care unit (ICU) stay of > 48 h were eligible for inclusion. Exclusion criteria included (1) transfer from other ICUs or hospitals; (2) treatment with antibiotics, prebiotics or probiotics before the first sample was obtained; (3) inability to obtain the first faecal sample within 72 h after admission; (4) use of antibiotics or admission to hospitals within 6 months prior to participation in the current study; and concomitant (5) gastrointestinal disease, (6) malignant cancer or (7) pregnancy. GCS, Acute Physiology and Chronic Health Evaluation (APACHE-II) and Sequential Organ Failure Assessment (SOFA) scores were recorded by specialized neurocritical care physicians at admission. The modified Rankin Scale (mRS) was recorded by specialized neurocritical care physicians at discharge. The healthy subjects were recruited from a community (the Bureau of Reclamation in Guangzhou) between November 2016 and January 2017. The volunteers who met the following inclusion criteria were recruited: (1) self-reported as healthy; (2) had no cross-border tourism recently; (3) had not taken antibiotics, prebiotics or probiotics within 1 year; and (4) had not been admitted to hospitals within 1 year prior to participation in the current study. Age- and sex-matching processes were further performed to select comparable controls. Written informed consent was obtained from all healthy subjects and patients or their legal representatives. Ethical approval for both the patients and healthy subjects was received from the Medical Ethics Committee of Nanfang Hospital (No. NFEC-2018-034), and all studies were conducted in accordance with the Declaration of Helsinki.

### Faecal sample collection and DNA extraction

The faecal samples were obtained from the patients within 72 h after admission and serially every week and were collected once from individuals in the control group. Fresh stool samples were frozen at − 80 °C within 3 h after voiding, and 0.2 g of each sample was aliquoted for DNA extraction. Bacterial DNA was extracted with a stool DNA extraction kit using a magnetic bead-based method (Shenzhen Bioeasy Biotechnology Co., Ltd., China) according to the manufacturer’s instructions [17].

### Polymerase chain reaction amplification of bacterial 16S rRNA genes

The V4 region of the bacterial 16S rRNA gene was amplified by quantitative real-time polymerase chain reaction (qPCR) with the bar-coded primers V4F (5′-GTGTGYCAGCMGCCGCGGTAA-3′) and V4R (5′-CCGGACTACNVGGGTWTCTAAT-3′) using a LightCycler 480 II real-time fluorescence quantitative PCR system (Roche Diagnostics Ltd., Switzerland). The qPCR protocol was as follows: (1) initial denaturation (94 °C, 2 min); (2) PCR amplification (32 cycles, 94 °C, 30 s; 52 °C, 30 s; followed by 72 °C, 30 s); (3) melting (95 °C, 5 s and 60 °C, 1 min; followed by 95 °C, continuous); and (4) cooling (37 °C, 30 s). Agarose gel electrophoresis (1%) was used to detect the PCR products. Samples that produced a visible product 290–310 bp in length were used for further experiments. GeneTools analysis software (version 4.03.05.0, SynGene) was used. The PCR products were mixed in equimolar ratios and purified using an EZNA Gel Extraction Kit (Omega, USA). Sequencing libraries were established using an NEBNext Ultra™ DNA Library Prep Kit for Illumina (New England Biolabs, USA) according to the manufacturer’s recommendations, and the index codes were added. The library quality was assessed using the Qubit@ 2.0 Fluorometer (Thermo Scientific) and Agilent Bioanalyzer 2100 systems. Finally, the library was sequenced on an Illumina HiSeq 2500 platform, and 250-bp paired-end reads were generated.

### Sequencing and microbial analysis

Sequences longer than 200 bp were trimmed to 200 bp, and those shorter than 200 bp were removed. Sequencing 200 bp at one end of each fragment had the advantages of a good overlap effect, minimal use of computing resources and improved sequence quality [18, 19]. Depending on the overlap between the two paired-end sequences, we then used SeqPrep to merge the paired-end sequences and assessed the quality of the result using the open-source software Quantitative Insights into Microbial Ecology (QIIME, version 1.9.1) [20]. A QIIME workflow script, split_libraries_fastq.py, was used to check the quality of the sequences, and sequences with a Phred score of ≥ Q20 were considered qualified sequences. Then, we used a home-brewed script to split FASTA files according to the paired-end barcode information, which met the following criteria: a 100% match between the barcode and the primer and more than 200 bp remaining after the removal of the barcode and primer. Finally, we used a QIIME workflow script, pick_closed_reference_otus.py, to remove chimaeras, perform reference-based operational taxonomic unit (OTU) clustering and generate a BIOM file. We used Greengenes 13.8 as the reference database and SortMeRNA for clustering and classification with an identity cut-off value of 0.97. All samples were normalized to the same level to avoid possible errors stemming from the use of different sequencing depths. Each sample was normalized to 7000 sequences for subsequent analysis.

The α-diversity (the complexity within a community) was estimated by four indexes and calculated by QIIME [20]: (a) Chao1, based on the richness of the sample; (b) observed species, a direct measure of the species number; (c) Shannon, intended to represent the species abundance and evenness; and (d) phylogenetic diversity (PD)–whole tree, an alternate method that accounts for the phylogenetic differences between the species that comprise the community. The β-diversity (which estimates differences between microbial communities) was analysed using the Bray–Curtis distance and unweighted UniFrac distance approaches [21, 22]. To determine the significantly different taxa between two groups, linear discriminant analysis (LDA) coupled with effect size measurement (LEfSe) was performed using an online utility (http://huttenhower.sph.harvard.edu/galaxy) [23]. Significantly different bacteria with LDA scores of ≥ 2.0 or ≥ 3.5 were diagrammed on taxonomic bar plots.

### Statistical analysis

The continuous nonparametric data are presented as medians (interquartile ranges, IQRs) and were analysed using Mann–Whitney *U* or Wilcoxon tests. The continuous parametric data are presented as the means (standard deviations, SDs) and were analysed with Student’s *t* test. The categorical data are presented as numbers (percentages, %) and were analysed using chi-squared tests. For microbial analysis, QIIME was additionally performed using the Adonis test as previously described [24]. A univariate Cox proportional hazards model was first performed, and the candidate variables with a *p* value of < 0.05 were further included in the multivariate Cox regression model for adjustment. The candidate variables included demographic data (age and sex), comorbidities (diabetes, hypertension, heart failure, intracranial infection, intracranial hypertension, and pneumonia), serum markers (white blood cell count, creatinine, blood urea nitrogen, cystatin C, brain natriuretic peptide, and C-reactive protein), indicators of critical illness (GCS, APACHE-II and SOFA scores and the length of ICU stay), medical treatments (mechanical ventilation and enteral nutrition), the biodiversity of the microbiota (Shannon, PD–whole tree, Chao 1, observed species and Simpson indexes) and the *z*-scores of specific taxa abundances or their changes. The abundance changes (marked as “Δtaxon”, e.g., “ΔEnterobacteriaceae”) were calculated by subtracting the abundance of specific taxa in the first sample from the abundance of specific taxa in the second sample. The association between abundances and 180-day mortality was analysed based on the increase in the abundances per SD. Correlations between variables were determined with the Spearman’s rank correlation test. SPSS version 20 (Statistical Package for Social Sciences, Chicago, IL, USA) was used for statistical analysis. Two-tailed *p* values of < 0.05 were considered statistically significant. The figures were generated using R version 3.4.3 (https://www.r-project.org/).

## Results

### Patient demographics

**Fig. 1:**
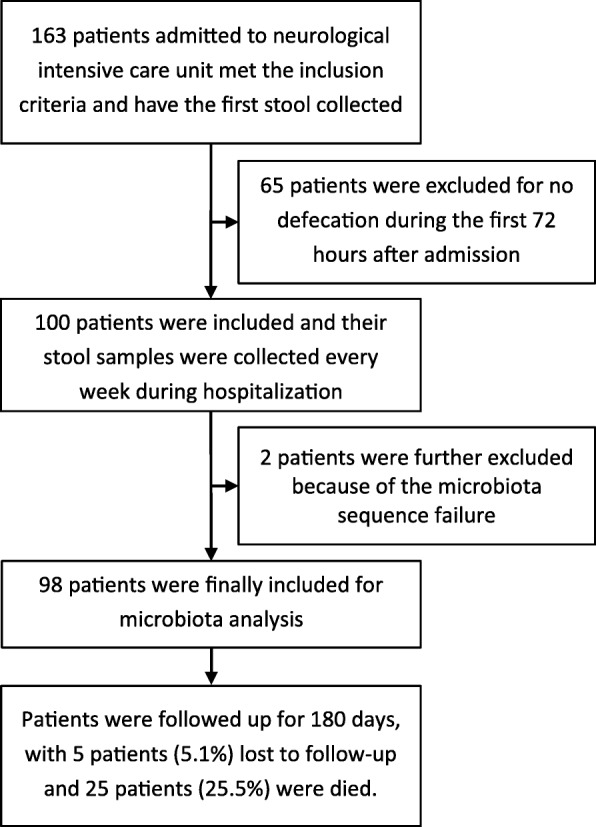
Flow diagram of the patient selection process

The first faecal samples were collected from 98 neurocritically ill patients (median age 58.5 years; 62.2% male) within 72 h after neuroICU admission. The flow diagram of the patient selection process is shown in Fig. 1. Of these 98 patients, 38 were admitted for ischaemic stroke, 20 for intracerebral haemorrhage, 13 for intracranial infection and 22 for seizure, hypoxic-ischaemic encephalopathy or another conditions (Additional file 3). Table 1 provides a summary of the characteristics of the study patients. In total, 206 faecal samples were collected for microbial analysis. These samples included 98 collected within 72 h after admission, 50 in the second week, 17 in the third week, 12 in the fourth week, 6 in the fifth week, 6 in the sixth week, 5 in the seventh week, 4 in the eighth week, 3 in the ninth week and 5 in the tenth to fourteenth weeks (Additional file 4). Eighty-four healthy subjects served as the healthy controls (HCs) and had faecal samples collected one time. The neuroICU group and HCs did not significantly differ in age (Mann–Whitney *U* test, *p* > 0.05) or sex (chi-squared test, *p* > 0.05) and did not differ in history of smoking, hypertension or diabetes (chi-squared test, *p* > 0.05) (Additional file 2: Table S1). The principal coordinate analysis (PCoA) plot did not show a significant difference in β-diversity (Bray–Curtis distance) between patients with different primary diagnoses, indicating that regardless of the primary diagnosis at admission, the gut microbiome of patients in the neuroICU differed substantially from that of healthy subjects (Additional file 2: Table S2; Additional file 1: Figure S1).

**Table 1:** Patients characteristics with neurocritically ill (*n* = 98)

Parameters	Value
Age (years), median (IQR)	58.5 (45–70.5)
Gender (male), *n* (%)	61 (62.2)
APACHE-II score, median (IQR)	13 (8–17.25)
GCS score, median (IQR)	7.5 (6–9)
SOFA score, median (IQR)	5 (3–7)
Main diagnosis, *n* (%)	
Ischaemic stroke	38 (38.78)
Intracerebral hemorrhage	20 (20.41)
Seizure	5 (5.1)
Intracranial infection	13 (13.27)
Hypoxic-ischaemic encephalopathy	5 (5.1)
Others	17 (17.35)
Comorbidities, *n* (%)	
Intracranial hypertension	25 (25.5)
Pneumonia	82 (83.7)
Intracranial Infection	15 (15.3)
Respiratory failure	20 (20.4)
Liver disease	39 (39.8)
Heart disease	40 (40.8)
Chronic renal disease	23 (23.5)
Hypertension	55 (56.1)
Diabetes mellitus	21 (21.4)
Patient history, *n* (%)	
Smoke exposure	34 (34.7)
Alcohol abuse	25 (25.5)
Treatment, *n* (%)	
Enteral nutrition	90 (91.8)
Mechanical ventilation	47 (48.0)
Clinical outcome, *n* (%)	
30-day survive	75 (76.5)
90-day survive	70 (71.4)
180-day survive	68 (69.4)

*APACHE-II* Acute Physiology and Chronic Health Evaluation-II, *GCS* Glasgow Coma Scale, *SOFA* Sequential Organ Failure Assessment

### Comparison of the gut microbiota characteristics in neurocritically ill patients and healthy subjects

**Fig. 2:**
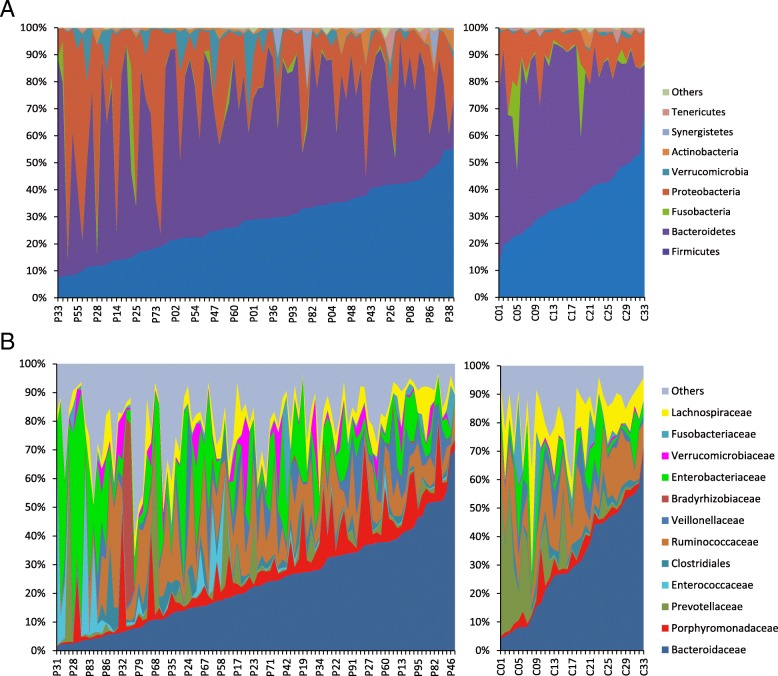
Area plot of the intestinal microbiota abundance of the first sample in each individual. The intestinal microbiota abundance was compared between neuroICU patients (P1, P2, …, P98) and HCs (C1, C2, …, C33) at phylum (**a**) and family (**b**) levels. *neuroICU* neurological intensive care unit, *HCs* healthy controls

We examined the first 98 faecal samples to explore the characteristics of the gut microbiota in the neuroICU patients (Additional file 1: Figure S2). The β-diversity and α-diversity between neuroICU patients and HCs were significantly different. As indicated by taxonomic summary and LEfSe analysis, the relative abundances of the phyla *Proteobacteria*, *Deferribacteres* and *Verrucomicrobia* were higher in the neuroICU group than in the HC group. At the family level, *Enterobacteriaceae*, *Porphyromonadaceae*, *Enterococcaceae*, *Verrucomicrobiaceae*, *Rikenellaceae* and *Lactobacillaceae* were enriched in the neuroICU group. To show the heterogeneity among individual gut microbiota compositions, we generated area plots from the first sample of each individual. At the phylum level, a high interindividual diversity in the faecal microbiota composition was observed in the neuroICU group (Fig. 2a). The faecal samples from patients P40 revealed a high abundance of *Verrucomicrobia* (> 30%), similar to the results reported by Lankelma et al. [25]. The bacterial composition analysis at the family level revealed an even greater interpatient diversity than that seen at the phylum level (Fig. 2b). *Enterococcaceae* comprised more than 70% of the microbiota in P31, and *Enterobacteriaceae* comprised more than 70% in two other patients (P53 and P64). However, none of the microbiomes in the HCs were dominated by any single pathogen to an extent of greater than 70%.

### Changes in the gut microbiota composition during neuroICU hospitalization

**Fig. 3:**
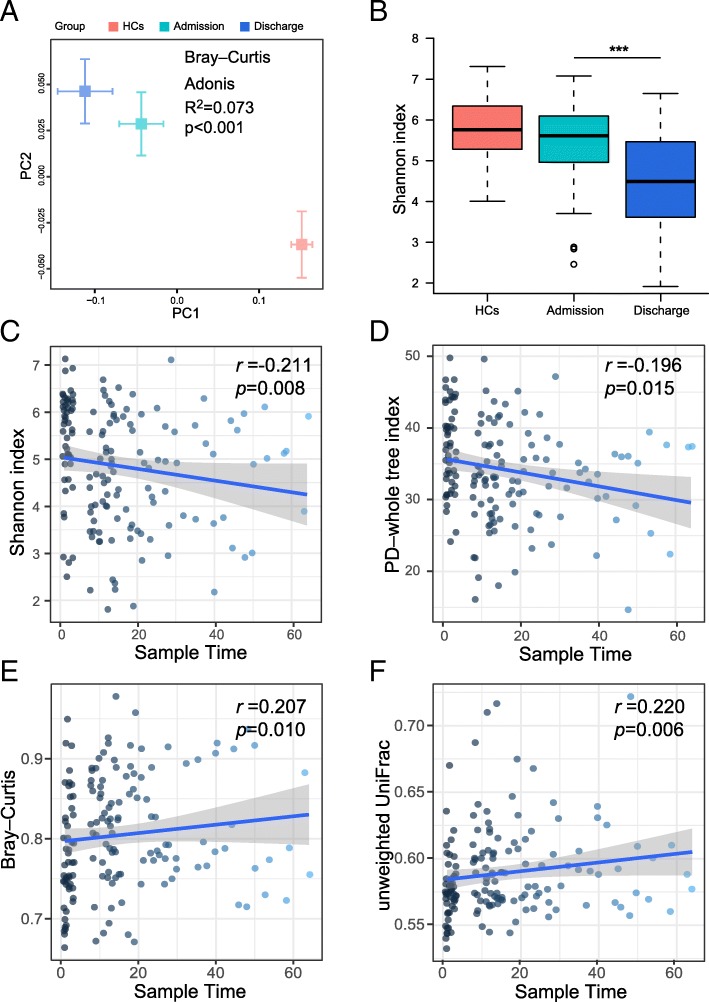
Dynamic changes in the intestinal microbiota of neuroICU patients. **a** PCoA plot illustrating the grouping patterns of the samples collected at admission and discharge. The “+” represents the mean and SD of the group. The distances between every “+” represent the dissimilarities between these two groups. **b** The diversity of the microbiota, presented as the Shannon index, was calculated from 50 paired samples collected within 72 h at admission and at discharge. ***, *p* < 0.001. **c**, **d** Temporal changes in the Shannon index (**c**) and PD–whole tree index (**d**) of the neuroICU group followed longitudinally every week during neuroICU hospitalization. **e**, **f** Temporal changes in the Bray–Curtis distance (**e**) and unweighted UniFrac distance (**f**) of the neuroICU group followed longitudinally every week during neuroICU hospitalization. *PCoA* principal coordinate analysis, *SD* standard deviation, *PD* phylogenetic diversity, *neuroICU* neurological intensive care unit

To examine the gut microbiota dynamics throughout the neuroICU stay, we next studied the community β-diversity in 50 patients from whom paired stool samples were obtained within 72 h after admission and at discharge. The other 48 patients were excluded, as they had only one available stool sample during their hospital stay. The bacterial community composition in all of these patients exhibited a substantial compositional divergence at discharge relative to that at admission (Fig. 3a). The samples collected at admission exhibited significantly higher community composition diversity (as indicated by the Shannon index) than the samples collected at discharge (Wilcoxon test, *p* < 0.001) (Fig. 3b). The microbiota changed radically after neuroICU admission. The α-diversities of the samples, which included only samples collected more than once from a single patient, were correlated inversely with the number of days spent in the neuroICU (*r* = − 0.211, *p* = 0.008; *r* = − 0.196, *p* = 0.015, respectively) (Fig. 3c, d). The distances between each sample in the neuroICU and HC groups were calculated by the Bray–Curtis and unweighted UniFrac metrics, which were plotted against the length of hospital stay. The Bray–Curtis distances and unweighted UniFrac were correlated positively with the number of days spent in the neuroICU (*r* = 0.207, *p* = 0.010; *r* = 0.220, *p* = 0.006, respectively) (Fig. 3e, f). The α-diversities changed greatly in the serial samples collected from the same patients several weeks apart (Additional file 1: Figure S3). In some subjects, the α-diversity recovered by the end of hospitalization (P01, P05 and P26), which was similar to a previous report [26].

**Fig. 4:**
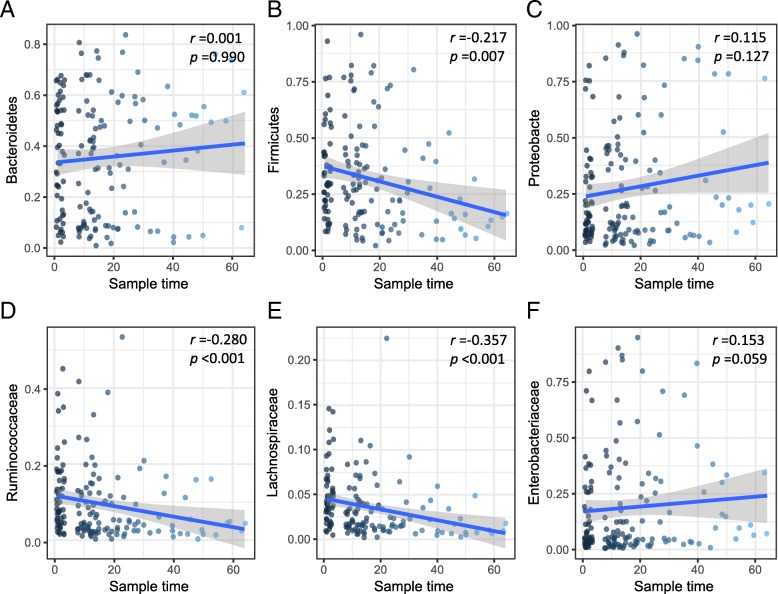
Dynamic changes in specific taxa during neuroICU hospitalization. *Bacteroidetes* (**a**) and *Proteobacteria* (**c**) were not correlated with the length of hospital stay. *Firmicutes* (**b**) was negatively associated with the sample collection time. *Ruminococcaceae* (**d**) and *Lachnospiraceae* (**e**) were significantly correlated with length of stay. *Enterobacteriaceae* (**f**) was not associated with the sample collection time

The correlation analysis between six high-abundance taxa and sample time is shown in Fig. 4. At the phylum level, *Bacteroidetes* and *Proteobacteria* were not correlated with the length of hospital stay (*r* = 0.001, *p* = 0.990; *r* = 0.115, *p* = 0.127, respectively). In addition, *Firmicutes* was negatively associated with the sample collection time (*r* = − 0.217, *p* = 0.007). *Ruminococcaceae* and *Lachnospiraceae* were significantly correlated with length of stay (*r* = − 0.280, *p* < 0.001; *r* = − 0.357, *p* < 0.001, respectively). At the family level, the correlation between *Enterobacteriaceae* and the length of stay exhibited a trend towards significance (*r* = 0.153, *p* = 0.059).

To explore the correlation between health status and intestinal microbiota, we further performed Spearman correlation analyses in patients who survived at discharge (*n* = 79), which showed that the increases in *Proteobacteria* (*r* = 0.254, *p* = 0.012), *Gammaproteobacteria* (*r* = 0.203, *p* = 0.045) and *Enterobacteriaceae* (*r* = 0.240, *p* = 0.017) were paralleled with the increase in mRS scores assessed at discharge.

### Associations between the abundance of intestinal microbiota and 180-day mortality

During the follow-up period, 25 (25.5%) patients died within 180 days of admission. A Cox proportional hazards regression model was constructed to examine the associations between abundances of specific taxa and 180-day mortality after ICU admission. The results of the univariate model showed that *Christensenellaceae* and *Erysipelotrichaceae* were associated with 180-day mortality and were independently associated with 180-day mortality in the multivariate Cox regression analysis adjusted for age, APACHE-II, white blood cell count and serum creatinine (adjusted hazard ratio (aHR) = 1.545; 95% confidence interval (CI) 1.163–2.053, *p* = 0.003; and aHR = 1.493; 95% CI, 1.094–2.038, *p* = 0.012, respectively) (Table 2).

**Table 2:** Cox regression analysis of risk factors associated with 180-day mortality in neurocritically ill patients (*n* = 98)

Parameters	Univariate		Multivariate	
	HR (95%CI)	*p* value	aHR (95%CI)	*p* value
Candidate variables				
Age	1.027 (1.004–1.051)	0.024		
APACHE-II	1.074 (1.005–1.146)	0.034		
WBC	1.091 (1.002–1.188)	0.045		
Cr	1.002 (1.000–1.004)	0.013		
The abundance of specific taxa of the first sample (*z*-score)				
*Christensenellacea*e	1.429 (1.080–1.892)	0.013	1.545* (1.163–2.053)	0.003
*Erysipelotrichaceae*	1.398 (1.029–1.900)	0.032	1.493* (1.094–2.038)	0.012

*Adjusted for age, APACHE-II, white blood cell count and serum creatinine

*HR* hazard ratio, *aHR* adjusted hazard ratio, *95%CI* 95% confidence interval, *SOFA* Sequential Organ Failure Assessment, *APACHE-II* Acute Physiology and Chronic Health Evaluation-II, *WBC* white blood cell count, *Cr* creatinine

Then, the association between the microbiota abundance changes and 180-day mortality after ICU admission was analysed by a Cox regression model. Fifty patients with paired samples both in the first 72 h and in the second week were included. The other 48 patients were excluded as they had only one available stool sample during their hospital stay. The results of the univariate model showed that the abundance changes in *Fusobacteria*, *Fusobacteriales*, *Enterobacteriales*, *Veillonellaceae*, *Fusobacteriaceae* and *Enterobacteriaceae* were associated with the 180-day mortality (Table 3). However, only increases in *Enterobacteriales* and *Enterobacteriaceae* were found to be independently associated with 180-day mortality in the multivariate Cox regression analysis adjusted for APACHE-II, respiratory failure, intracranial hypertension and serum lactate (Table 3). The increases in *Enterobacteriales* and *Enterobacteriaceae* during the first week in the neuroICU were both associated with increases of 92% in the risk of 180-day mortality after adjustments.

**Table 3:** Cox regression analysis of risk factors associated with 180-day mortality in neurocritically ill patients (*n* = 50)

Parameters	Univariate		Multivariate	
	HR (95%CI)	*p* value	aHR (95%CI)	*p* value
Candidate variables				
APACHE-II	1.299 (1.008–1.674)	0.044		
Respiratory failure	3.853 (1.033–14.366)	0.045		
Intracranial hypertension	4.472 (1.115–17.938)	0.035		
Serum lactate	1.367 (1.042–1.793)	0.024		
The changes of the specific taxon abundance during first week after admission (*z*-score)				
Δ*Fusobacteria*	0.262 (0.097–0.708)	0.008	–	NS
Δ*Fusobacteriales*	0.262 (0.097–0.708)	0.008	–	NS
Δ*Enterobacteriales*	1.756 (1.010–3.056)	0.046	1.920^♯^ (1.016–3.628)	0.044
Δ*Veillonellaceae*	1.475 (1.005–2.165)	0.047	–	NS
Δ*Fusobacteriaceae*	0.262 (0.097–0.706)	0.008	–	NS
Δ*Enterobacteriacea*e	1.756 (1.010–3.056)	0.046	1.920^♯^ (1.016–3.628)	0.044

^♯^Adjusted for APACHE-II, respiratory failure, intracranial hypertension and serum lactate

*HR* Hazard ratio, *aHR* adjusted hazard ratio, *95%CI* 95% confidence interval, *SOFA* Sequential Organ Failure Assessment, *APACHE-II* Acute Physiology and Chronic Health Evaluation-II, *WBC* white blood cell, *Cr* creatinine, *NS* not significant

### Subgroup analysis

To eliminate confounding from the primary diagnosis and the differences in pathophysiology, we performed a subgroup analysis in patients with critically ill stroke, including 58 patients diagnosed with ischaemic stroke and intracerebral haemorrhage (median age 63 years; 60.3% male; median NIHSS score 12) (Table 4). Fifty-eight age- and sex-matched healthy subjects were selected as HCs (Additional file 2: Table S3). The characteristics of the gut microbiota in patients with stroke were significantly different from those in healthy subjects (Additional file 1: Figure S4), as indicated by the β-diversity, α-diversity, taxonomic summary and LEfSe analysis.

**Table 4:** Patients characteristics with critically ill stroke (*n* = 58)

Parameters	Value
Age (years), median (IQR)	63 (53.75–76.5)
Gender (male), *n* (%)	35 (60.3)
APACHE-II score, median (IQR)	13.5 (7.75–18.0)
GCS score, median (IQR)	8 (6–9.25)
SOFA score, median (IQR)	4 (3–7)
NIHSS score, median (IQR)	12 (10–17)
Comorbidities, *n* (%)	
Intracranial hypertension	10 (17.2)
Pneumonia	45 (77.6)
Intracranial Infection	2 (3.5)
Respiratory failure	3 (5.2)
Liver disease	19 (32.8)
Heart disease	28 (48.3)
Chronic renal disease	11 (19.0)
Hypertension	42 (72.4)
Diabetes mellitus	15 (25.9)
Patient history, *n* (%)	
Smoke exposure	21 (36.2)
Alcohol abuse	15 (25.9)
Treatment, *n* (%)	
Enteral nutrition	51 (87.9)
Mechanical ventilation	25 (43.1)
Clinical outcome, *n* (%)	
30-day survive	44 (75.9)
90-day survive	43 (74.1)
180-day survive	43 (74.1)

*APACHE-II* Acute Physiology and Chronic Health Evaluation-II, *GCS* Glasgow Coma Scale, *SOFA* Sequential Organ Failure Assessment, *NIHSS* National Institute of Health Stroke Scale

## Discussion

In this study, an in-depth faecal microbiota analysis was performed in neurocritically ill patients. We documented both changes in the diversities and large interindividual diversities in these patients relative to those characteristics in HCs. An overgrowth of opportunistic pathogens defined dysbiosis in patients with neurocritical illness. We also found that the bacterial community composition in the intestine at discharge significantly diverged from that observed at admission. In addition, the Cox regression analyses demonstrated that the abundances of *Christensenellaceae* and *Erysipelotrichaceae* at admission, as well as the abundance increase in *Enterobacteriaceae* during the ICU stay, were significantly associated with patients’ mortality within 180 days. To the best of our knowledge, this prospective observational study represents the largest cohort study of neurocritically ill patients to perform 16S rRNA gene sequence-based analyses of the gut microbiota and explore the association between the abundances of specific bacteria and survival status during the following 180 days in the setting of neurocritical illness.

Intestinal microbiota dysbiosis can be defined as an altered dialogue between the host’s cells and enteric bacteria due to disruption of microbial diversity usually manifested as the dominance of a given taxon. In recent decades, basic and translational studies have elucidated the functions of and architectural shifts in the intestinal microbiota in specific conditions and processes such as metabolic disease [27], central nervous system function [28, 29], systemic inflammation [30, 31] and immune function [32]. More recently, convincing evidence has emerged to report the appearance of pathogens, disappearance of commensals and loss of microbial diversity in the pathogenesis of critical illness [3, 9, 25, 26, 33–35]. We previously reported significant dysbiosis of the gut microbiota in patients with stroke and transient ischaemic attack [24] or chronic kidney disease [36]. Very recently, our research showed that participants at a high risk of stroke were characterized by the enrichment of opportunistic pathogens, low abundance of butyrate-producing bacteria and reduced concentrations of faecal butyrate [37]. As a whole, these investigations could contribute to an increased awareness among clinicians that disturbances in the gut microbiota might be associated with poor prognosis. Dysbiosis of the microbiota can be reasonably presumed to increase the risk of adverse events, including infection, undernutrition and unconsciousness. Consistent with the findings of prior studies, our results confirmed that the microbiome in patients in neurocritical care differed substantially from that in healthy subjects. For example, the samples from the neuroICU group were characterized by gut microbiota dysbiosis and a loss of health-promoting commensal microbes. These differences were further characterized using LEfSe, which showed that neuroICU patients tended to have lower relative abundances of *Firmicutes* and *Bacteroidetes* and an increased level of *Proteobacteria* than healthy subjects. This difference is unlikely to be a study effect, as the samples from patients were rigorously processed and run in parallel with those from healthy subjects. Moreover, we collected our samples in the same city to partially avoid geographical effects [19]. In addition, the disruption of the microbial community appeared to be greater at discharge than during the ICU hospitalization, with the decreased abundance of the butyrate-producing bacteria *Lachnospiraceae* and *Ruminococcaceae*. This ICU-acquired dysbiosis likely resulted from the pressures imposed by various patient-related factors and modern intensive care therapy. The use of antibiotics [38], vasoactive agents, artificial nutrition, proton pump inhibitors, analgesics or sedative agents that impair intestinal motility [39] may affect the gut microbiota. Antibiotic treatment precluded distinguishing the effect of critical illness from the effect of antibiotic pressure. However, the current study failed to address the impact of antibiotics on microbiota since most patients enrolled in this cohort received antimicrobial treatments, which is a common clinical practice in critical care. Because of the ubiquitous use of antibiotics worldwide [40], antibiotic pressure should be considered both a medical treatment and an inevitable pathophysiological insult that could exert negative effects on beneficial organisms.

Our findings that the abundances of *Christensenellaceae* and *Erysipelotrichaceae* are potential risk indicators of mortality within 180 days are consistent with previous reports. *Christensenellaceae* and *Erysipelotrichaceae* have been found to be associated with metabolic disorders and gastrointestinal diseases [41–43]. *Christensenellaceae* was reported to be enriched in patients with multiple sclerosis [44]. Some studies have shown that HIV-infected individuals [45] and individuals with non-alcoholic fatty liver disease [46] and Rett syndrome [47] exhibited a high abundance of *Erysipelotrichaceae*. Moreover, our results showed that the abundance increase in *Enterobacteriaceae* is associated with death within 180 days. As previously reported, *Enterobacteriaceae* is one of the most detrimental pathogens to humans, especially for patients in ICUs [48, 49]. Our results provide evidence potentially suggesting that high abundances of some specific pathogens or their abundance changes may contribute to poor clinical outcomes. However, due to the limited sample size, lack of confounder control and regional variations, the results from the Cox regression analysis are insufficient for worldwide generalization. Nonetheless, the relationship between the gut microbiota and poor prognosis might serve as a platform for identifying potential targets for mortality prediction in the future. Freedberg et al. [9] provided novel insights into the possibility of predicting death or subsequent infection in critically ill patients by the characteristics of the gut microbiota upon ICU admission. Both colonization with vancomycin-resistant enterococci and the dominance of *Enterococcus* species were associated with death or all-cause infection adjusted by illness severity. Some studies have suggested that critical illness can lead to translocation of gut microbes into the bloodstream [50, 51], potentially explaining the association between specific pathogens and mortality. Identifying the critically ill patients most at risk of mortality is crucial for medical treatment strategies and doctor-patient communication. An intriguing possibility is analysing stool samples by 16S rRNA gene sequence as a non-invasive method for pretreatment assessment at admission to describe the role of the faecal “fingerprint” in predicting poor outcomes.

As the gut is hypothesized to play key roles in the progression of critical illness and multiple organ failure [4, 52], the restoration of commensal “healthy microbes” or eradication of pathogens might exert beneficial effects in critically ill patients. Microbiome intervention therapy includes antibiotic treatment, probiotic administration and faecal microbiota transplantation. Two case reports have discussed the successful application of faecal microbiota transplantation in patients with diarrhoea and therapy-resistant sepsis [53, 54]. However, these strategies should be implemented with personalized knowledge of the bacterial colonization patterns in the patients, as well as the selection of appropriately high-risk patients prior to implementing any intervention. Prior investigations of the ICU microbiome have generally been restricted by the limitations of culture-based approaches, which can detect only certain species of bacteria and cannot provide a complete picture immediately during acute illness. The 16S rRNA gene sequence technique has allowed clinicians to perform sequence-based investigations of microbes previously thought to be inaccessible [55]. This achievement may inspire the development of simple and effective microbiota-targeted therapies.

Our study has several strengths. This prospective observational study used 16S rRNA sequence analysis to identify members of the intestinal microbiota, exploring early samples after admission and longitudinal samples obtained weekly to provide dynamic observations. This research was the first to investigate the microbiota in neurocritically ill patients, with a correlation analysis between the abundances of taxa and clinical severity scores, as well as survival analyses based on the abundances of or changes in specific taxa and the rigorously adjudicated primary outcome of death. However, this research also has some inherent limitations that warrant further discussion. First, the window of our first sample enrolment was 72 h after admission, and the underlying changes in microbial diversity may occur during the period of the first sample collection. Although rectal swabs could obtain samples immediately and have been shown to provide similar efficacies as stool [56], faecal sampling was used in this study to avoid harmful effects on the patients. Therefore, the sample collection process was restricted to patients’ defecation cycle, and selection bias was present because faecal samples could be collected only from patients who defecated spontaneously. Second, the limited sample size and the substantial heterogeneity of the selected patients make the interpretation of the relationship between the microbiota composition and prognosis challenging. Third, the 16S rRNA gene sequence method used in this study could only evaluate the composition percentages but not the absolute numbers of microorganisms in the samples. An advanced technique is needed to define the gut microbiota more precisely. Finally, we could not continue to analyse faecal samples after discharge, and the recovery of the microbiome might be prolonged. We would like to evaluate post-discharge microbiota dynamics in future studies.

## Conclusions

In summary, we identified significant alterations in the gut microbiota composition between neurocritically ill patients and healthy subjects as well as marked dynamic changes in the patients’ gut microbiomes during hospitalization. The abundance and its changes were important risk factors for 180-day mortality, indicating that a gut microbiome examination could be a useful tool for identifying high-risk patients. High-quality prospective investigations with substantial samples collected on the day of admission and, ideally, with concurrent experimental models, are warranted to confirm the findings of this research and evaluate the net effect of microbiota dysbiosis on the progression and outcome of neurocritical illness.

## Additional files

Additional file 1: **Figure S1.** PCoA plot illustrating the grouping patterns of the samples collected from patients with different primary diagnoses at admission. **Figure S2.** The gut microbiota composition of neuroICU patients was significantly different from that of 84 healthy subjects. **Figure S3.** Dynamic changes in α-diversities of samples from seven patients with neuroICU stay lengths of greater than six weeks. **Figure S4.** The gut microbiota composition of 58 patients with critically ill stroke was significantly different from that of 58 healthy subjects. (DOCX 1152 kb)

Additional file 2: **Table S1.** Baseline characteristics of the patients from the neuroICU and the healthy subjects. **Table S2.** Comparison of β-diversity between patients with different primary diagnoses and healthy controls. **Table S3.** Baseline characteristics of patients with critically ill stroke and the healthy subjects. (DOCX 25 kb)

Additional file 3: Clinical information of all patients in the neuroICU group in this study. (XLSX 25 kb)

Additional file 4: α-Diversities and taxa of all samples collected from neuroICU and HCs in this study. (XLSX 922 kb)

## Data Availability

The datasets used and/or analysed during the current study are available from the corresponding author on reasonable request.

## Abbreviations

aHR: Adjusted hazard ratio
APACHE-II: Acute Physiology and Chronic Health Evaluation-II
CI: Confidence interval
GCS: Glasgow Coma Scale
HCs: Healthy controls
HR: Hazard ratio
ICU: Intensive care unit
IQR: Interquartile range
LDA: Linear discriminant analysis
LEfSe: Linear discriminant analysis effect size
mRS: Modified Rankin Scale
neuroICU: Neurological intensive care unit
OTU: Operational taxonomic unit
PCoA: Principal coordinate analysis
PD: Phylogenetic diversity
QIIME: Quantitative Insights into Microbial Ecology
qPCR: Quantitative real-time polymerase chain reaction
SD: Standard deviation
SOFA: Sequential Organ Failure Assessment
