# Supplementary material for: Dysbiosis of the intestinal microbiota in neurocritically ill patients and the risk for death
Source: Crit Care. 2019 May 31;23:195. doi: 10.1186/s13054-019-2488-4 (PMC6544929; doi:10.1186/s13054-019-2488-4)

**Supplementary Figures**

**Figure S1. PCoA plot illustrating the grouping patterns of the samples collected from patients with different primary diagnoses at admission.** (A) Bray-Curtis distance, R^2^=0.084, p<0.001. (B) unweighted UniFrac distance, R^2^=0.090, p<0.001. The “＋” represents the mean and SD of the group. The distances between every “＋” represent the dissimilarities between these two groups. *PCoA* Principal coordinate analysis, *HCs* healthy controls, *IS* ischaemic stroke, *ICH* intracerebral haemorrhage, *CNS* central nervous system, *HIE* hypoxic-ischaemic encephalopathy


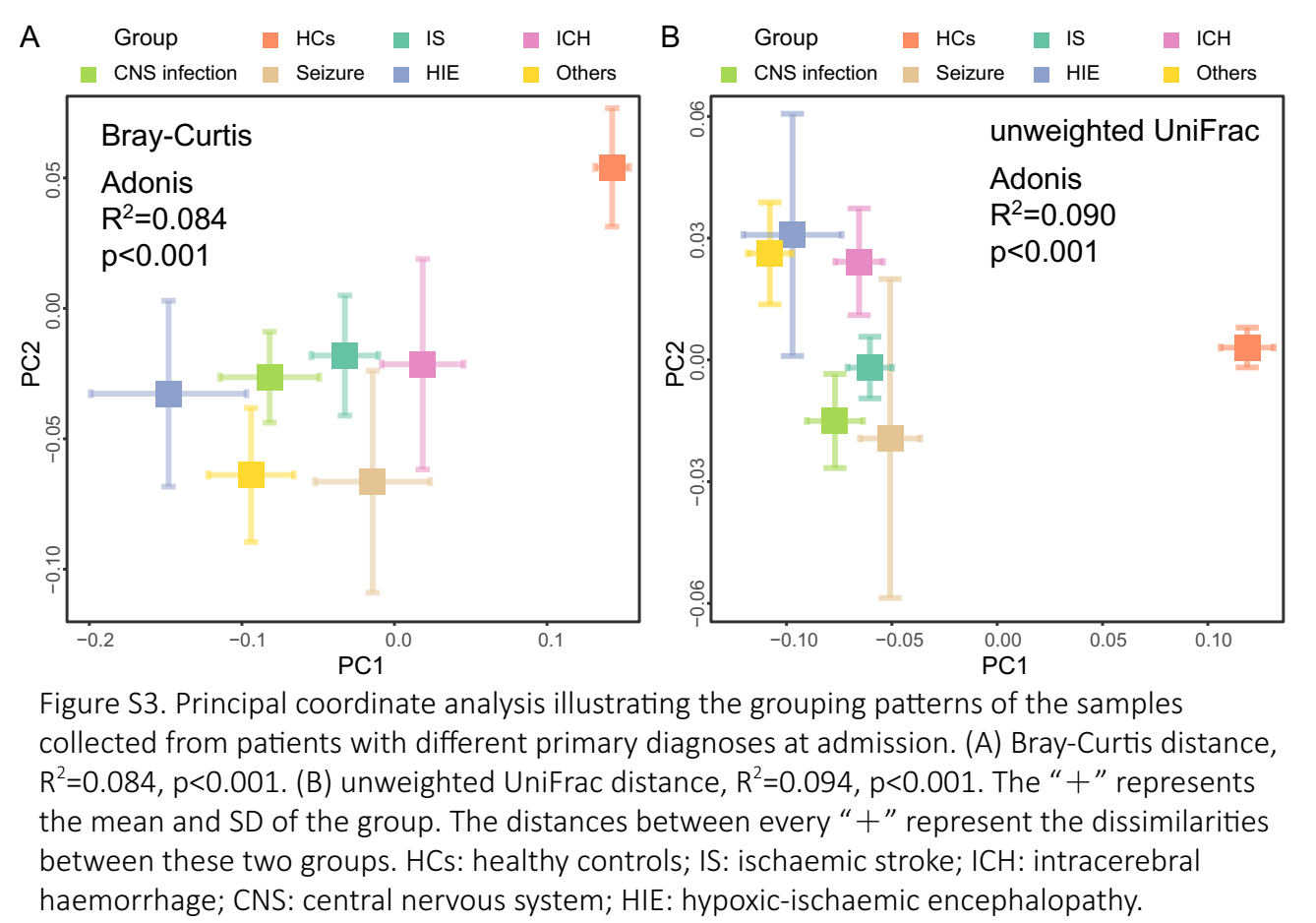


**Figure S2. The gut microbiota composition of neuroICU patients was significantly different from that of 84 healthy subjects.** (A) The β diversity in the neuroICU and HCs groups was calculated by the Bray–Curtis distance and is shown in the PCoA plot. Each point represents the composition of the intestinal microbiota of one participant. (B) The diversity of the microbiota, presented as the Shannon index, was calculated from samples from 98 neuroICU patients and 84 HCs (Mann–Whitney U test, p<0.001). The boxplots display the 95% CIs, and the points lying outside the whiskers are referred to as outliers. ***, p<0.001. (C, D) Average relative abundances of the predominant bacterial taxa at the phylum (C) and family (D) levels in neuroICU patients and HCs. (E, F) Significantly discriminative taxa between the neuroICU and HCs groups were determined using linear discriminant analysis effect size at the phylum(threshold=3.0) (E) and family (threshold=3.5) (F) levels. The red bar chart represents the bacteria that were more abundant in the faecal samples from the patients, and the blue bar chart represents those more abundant in the faecal samples from the HCs. *neuroICU* neurological intensive care unit, *HCs* healthy controls, *PCoA* Principal coordinate analysis, *CI* confidence interval


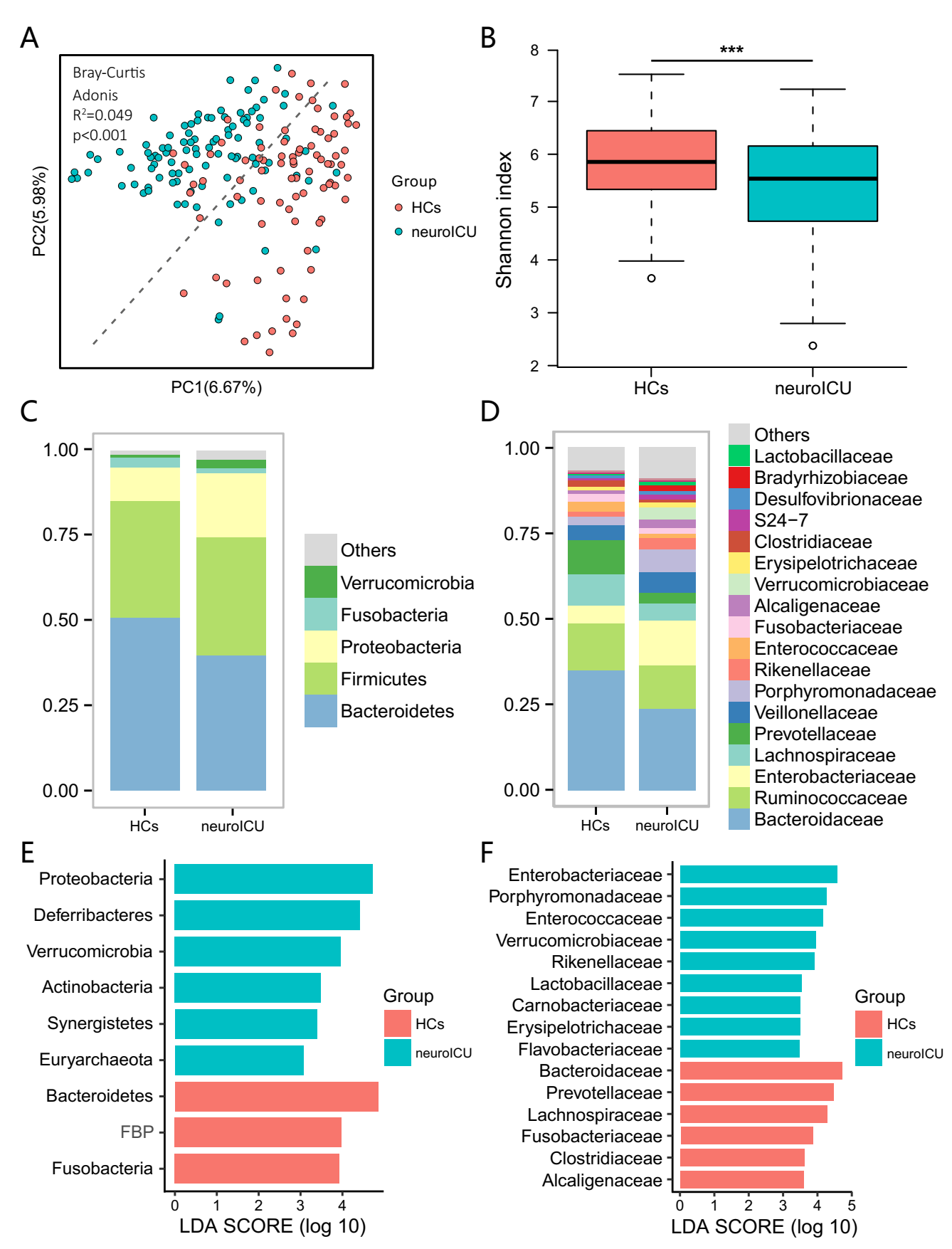


**Figure S3. Dynamic changes in α diversities of samples from seven patients with neuroICU stay lengths of greater than six weeks.** (A) the Shannon index. (B) the PD–whole tree index. *neuroICU* neurological intensive care unit


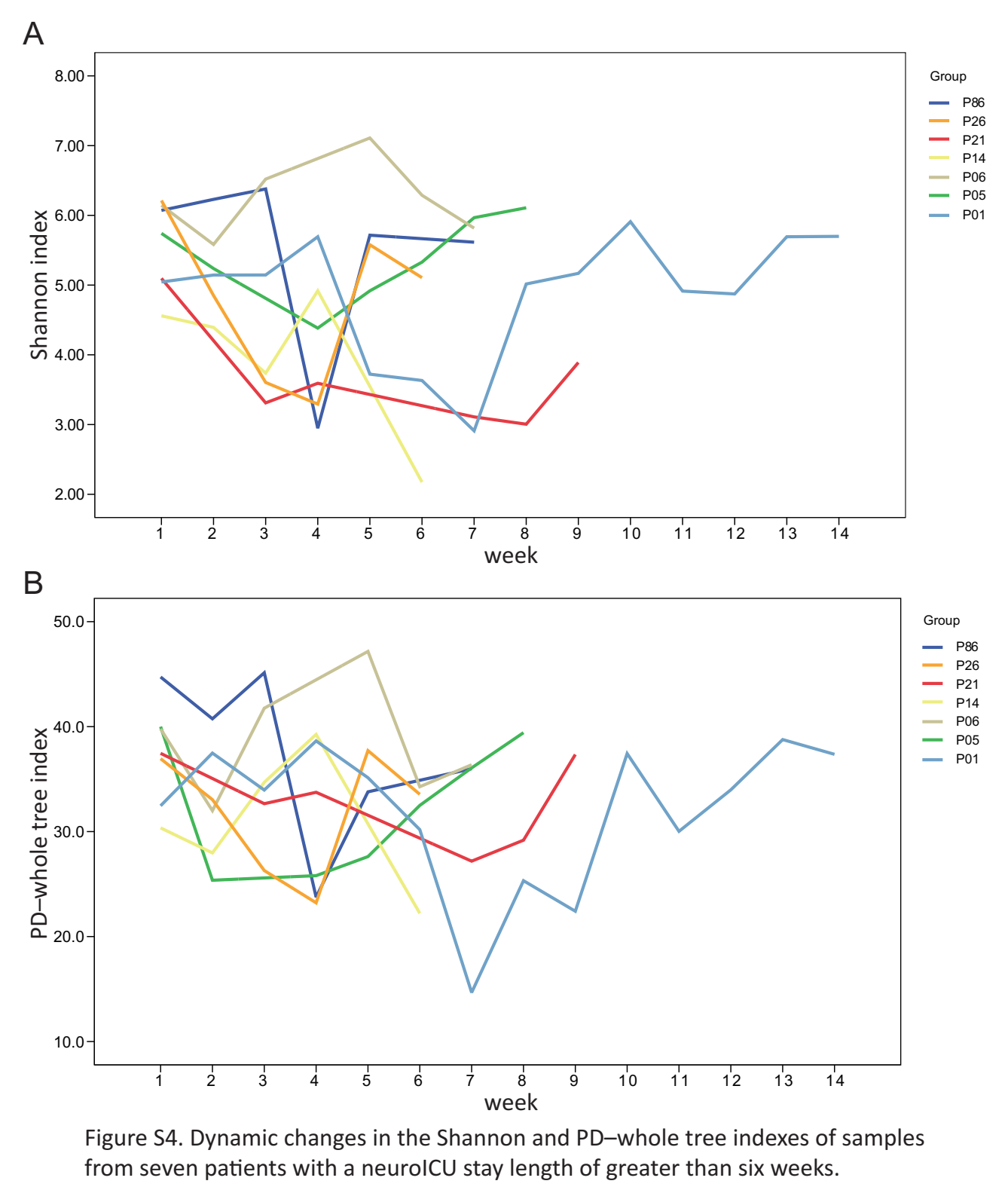


**Figure S4. The gut microbiota compositions of 58 patients with critcally ill stroke were significantly different from that of 58 healthy subjects.** (A) The β diversity in the stroke and HC groups was calculated by the Bray–Curtis distance and is shown in the PCoA plot. Each point represents the composition of the intestinal microbiota of one participant. (B) The diversity of the microbiota, presented as the Shannon index, was calculated from samples from 58 patients with critcally ill stroke and 58 healthy subjects (Mann-Whitney U test, *p*=0.016). The boxplots display the 95% CIs, and the points lying outside the whiskers are referred to as outliers. *, p<0.001. (C, D) Average relative abundances of the predominant bacterial taxa at the phylum level and the family level in patients with critcally ill stroke and healthy subjects. (E, F) Significantly discriminative taxa between the control participants and patients with critcally ill stroke were determined using linear discriminant analysis effect size at the phylum (threshold=2.0) (E) and family (threshold=3.5) (F) levels. The red bar chart represents the bacteria that were more abundant in the faecal samples from the patients, and the blue bar chart represents those that were more abundant in the faecal samples from the HCs. *neuroICU* neurological intensive care unit, *HCs* healthy controls, *PCoA* Principal coordinate analysis, *CI* confidence interval


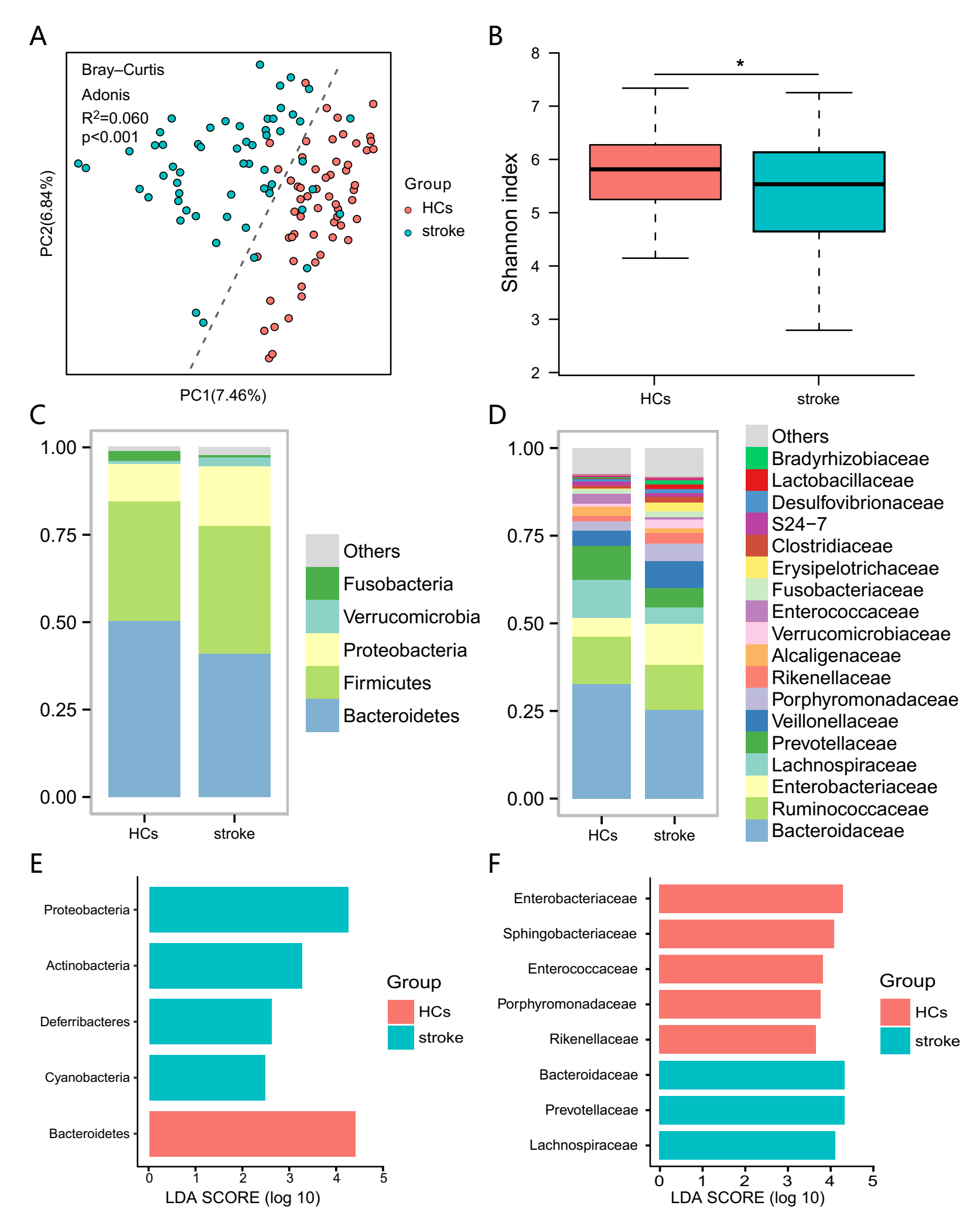

Supplement: Supplementary file 1 — Figure S1. PCoA plot illustrating the grouping patterns of the samples collected from patients with different primary diagnoses at admission. Figure S2. The gut microbiota composition of neuroICU patients was significantly different from that of 84 healthy subjects. Figure S3. Dynamic changes in α-diversities of samples from seven patients with neuroICU stay lengths of greater than six weeks. Figure S4. The gut microbiota composition of 58 patients with critically ill stroke was significantly different from that of 58 healthy subjects. (DOCX 1152 kb) [file 13054_2019_2488_MOESM1_ESM.docx]
