# Supplementary material for: Dysbiosis of the intestinal microbiota in neurocritically ill patients and the risk for death
Source: Crit Care. 2019 May 31;23:195. doi: 10.1186/s13054-019-2488-4 (PMC6544929; doi:10.1186/s13054-019-2488-4)
Supplement: Supplementary file 2 — Table S1. Baseline characteristics of the patients from the neuroICU and the healthy subjects. Table S2. Comparison of β-diversity between patients with different primary diagnoses and healthy controls. Table S3. Baseline characteristics of patients with critically ill stroke and the healthy subjects. (DOCX 25 kb) [file 13054_2019_2488_MOESM2_ESM.docx]

**Supplementary Tables**

**Table S1** Baseline characteristics of the patients from the neuroICU and the healthy subjects.

| Parameters | neuroICU(*n*=98) | HCs(*n*=84) | *p* value |
| --- | --- | --- | --- |
| Age (years), median (IQR) | 58.5(45-70.5) | 58(52-68.75) | 0.588 |
| Gender (male), n (%) | 61(62.2) | 48(57.1) | 0.484 |
| Smoke (yes), n (%) | 34(34.7) | 40(47.6) | 0.077 |
| Hypertension (yes), n (%) | 55(56.1) | 53(63.1) | 0.340 |
| Diabetes (yes), n (%) | 21(21.4) | 18(21.4) | 1.000 |
| Serum markers, median (IQR) |  |  |  |
| WBC, ×10^9^ | 9.54 (7.80-13.10) | 5.99 (5.05-7.13) | <0.001 |
| NEU, ×10^9^ | 7.86 (5.74-10.51) | 3.15 (2.56-4.07) | <0.001 |
| RBC, ×10^12^ | 4.33 (3.64-4.89) | 4.7 (4.39-5.16) | <0.001 |
| HGB, g/L | 127.5 (108-145.25) | 137 (127.25-148) | <0.001 |
| PLT, ×10^12^ | 201 (163.75-258) | 244.5 (211-281.75) | <0.001 |
| TP, g/L | 65.8 (61.08-70.75) | 71.85 (69.7-74.08) | <0.001 |
| ALB, g/L | 37.1 (32.28-41.1) | 42.3 (40.5-43.48) | <0.001 |
| HDL, mmol/L | 0.83 (0.39-1.06) | 1.31 (1.16-1.59) | <0.001 |
| LDL, mmol/L | 2.18 (1.21-3.24) | 3.29 (2.8-3.93) | <0.001 |
| TG, mmol/L | 0.96 (0.63-1.41) | 1.23 (0.88-1.73) | 0.001 |
| VLDL, mmol/L | 0.42 (0.12-0.78) | 0.65 (0.49-0.76) | 0.001 |
| BUN, mmol/L | 6.0 (4.2-7.95) | 5.0 (4.5-5.48) | 0.002 |
| Cr, μmol/L | 75 (54-98) | 63.5 (55.25-79.75) | 0.023 |
| Glu, mmol/L | 6.6 (5.41-8.43) | 4.78 (4.4-5.2) | <0.001 |
| TC, mmol/L | 23.35 (21.3-25) | 5.46 (4.61-6) | <0.001 |
| *WBC* white blood cells, *NEU* neutrophils, *RBC* red blood cells, *HGB* hemoglobin, *PLT* platelet, *TP* total protein, *ALB* albumin, *HDL* high density lipoprotein, *LDL* low density lipoprotein, *TG* triglyceride, *VLDL* very low density lipoprotein, *BUN* blood urea nitrogen, *Cr* creatinine, *Glu* glucose, *TC* total cholesterol | | | |

**Table S2** Comparison of β diversity between patients with different primary diagnoses and healthy controls.

| Bray–Curtis | IS | ICH | CNS infection | seizure | HIE | others |
| --- | --- | --- | --- | --- | --- | --- |
| IS | – | – | – | – | – | – |
| ICH | 0.110 | – | – | – | – | – |
| CNS infection | 0.675 | 0.062 | – | – | – | – |
| seizure | 0.935 | 0.412 | 0.861 | – | – | – |
| HIE | 0.097 | 0.067 | 0.503 | 0.453 | – | – |
| others | 0.096 | 0.039 | 0.447 | 0.595 | 0.279 | – |
| HCs | <0.001 | <0.001 | <0.001 | 0.044 | <0.001 | <0.001 |
| unweighted UniFrac | IS | ICH | CNS infection | seizure | HIE | others |
| IS | – | – | – | – | – | – |
| ICH | 0.166 | – | – | – | – | – |
| CNS infection | 0.176 | 0.270 | – | – | – | – |
| seizure | 0.239 | 0.187 | 0.587 | – | – | – |
| HIE | 0.090 | 0.398 | 0.716 | 0.501 | – | – |
| others | 0.041 | 0.088 | 0.044 | 0.157 | 0.216 | – |
| HCs | <0.001 | <0.001 | <0.001 | <0.001 | <0.001 | <0.001 |

*HCs* healthy controls, *IS* ischemic stroke, *ICH* intracerebral hemorrhage, *CNS* central nervous system, *HIE* hypoxic-ischemic encephalopathy

**Table S3** Baseline characteristics of the patients with critically ill stroke and the healthy subjects.

| Parameters | stroke (n=58) | HC (n=58) | p value |
| --- | --- | --- | --- |
| Age (years), median (IQR) | 63 (53.75-76.5) | 61.5 (57.75-73.5) | 0.667 |
| Gender (male), n (%) | 35 (60.3) | 33 (59.6) | 0.706 |
| Smoke | 21 (36.2) | 31 (53.4) | 0.062 |
| Hypertension | 42 (72.4) | 41 (70.7) | 0.837 |
| Diabetes | 15 (25.9) | 13 (22.4) | 0.664 |
| Serum markers, median (IQR) | |  |  |
| WBC, ×10^9^ | 9.69 (7.87-12.96) | 6.05 (5-7.21) | <0.001 |
| NEU, ×10^9^ | 7.98 (5.69-10.99) | 3.25 (2.57-4.11) | <0.001 |
| RBC, ×10^12^ | 4.57 (3.9-4.94) | 4.66 (4.38-5.03) | 0.028 |
| HGB, g/L | 130 (112-147) | 139.5 (128.5-151) | 0.011 |
| PLT, ×10^12^ | 200.5 (167-258.5) | 224 (205-266) | 0.006 |
| TP, g/L | 65.7 (62.83-71.08) | 71.55 (69.38-73.58) | <0.001 |
| ALB, g/L | 38.4 (35.45-41.93) | 41.85 (40.08-43.2) | <0.001 |
| HDL, mmol/L | 0.91 (0.77-1.19) | 1.3 (1.19-1.58) | <0.001 |
| LDL, mmol/L | 2.65 (1.76-3.45) | 3.2 (2.76-3.86) | <0.001 |
| TG, mmol/L | 1.02 (0.73-1.38) | 1.25 (0.87-1.72) | 0.021 |
| VLDL, mmol/L | 0.55 (0.26-0.82) | 0.65 (0.51-0.75) | 0.063 |
| BUN, mmol/L | 6 (4.28-7.35) | 5 (4.48-5.63) | 0.013 |
| Cr, μmol/L | 84.5 (64.75-100) | 73 (58.75-88) | 0.008 |
| Glu, mmol/L | 7.1 (5.43-8.75) | 4.74 (4.4-5.17) | <0.001 |
| TC, mmol/L | 23.2 (21.3-24.63) | 5.42 (4.5-5.99) | <0.001 |
| *WBC* white blood cells, *NEU* neutrophils, *RBC* red blood cells, *HGB* hemoglobin, *PLT* platelet, *TP* total protein, *ALB* albumin, *HDL* high density lipoprotein, *LDL* low density lipoprotein, *TG* triglyceride, *VLDL* very low density lipoprotein, *BUN* blood urea nitrogen, *Cr* creatinine, *Glu* glucose, *TC* total cholesterol | | | |
